# Short-term Beneficial Effects of 12 Sessions of Neurofeedback on Avoidant Personality Accentuation in the Treatment of Alcohol Use Disorder

**DOI:** 10.3389/fpsyg.2017.01688

**Published:** 2017-09-26

**Authors:** Nina Dalkner, Human F. Unterrainer, Guilherme Wood, Dimitris Skliris, Sandra J. Holasek, John H. Gruzelier, Christa Neuper

**Affiliations:** ^1^Institute of Psychology, Karl-Franzens University of Graz, Graz, Austria; ^2^Department of Psychiatry and Psychotherapeutic Medicine, Medical University of Graz, Graz, Austria; ^3^Center for Integrative Addiction Research (Grüner Kreis Society), Vienna, Austria; ^4^Institute of Pathophysiology and Immunology, Medical University of Graz, Graz, Austria; ^5^Department of Psychology, Goldsmiths, University of London, London, United Kingdom

**Keywords:** neurofeedback, alcohol use disorder, electroencephalography (EEG), treatment outcome, avoidant personality accentuation, Big Five

## Abstract

This study evaluated the effects of alpha/theta neurofeedback on Clinical Personality Accentuations in individuals with alcohol use disorder. Twenty-five males were investigated using a pre-test/post-test design with a waiting-list control group. Participants were randomly assigned either to an experimental group (*n* = 13) receiving 12 sessions of neurofeedback twice a week as a treatment adjunct over a period of 6 weeks, or to a control group (*n* = 12) receiving treatment as usual. The Inventory of Clinical Personality Accentuations and the NEO-Five-Factor Inventory were applied at pre- and post-test. The neurofeedback protocol focused on enhancement of the EEG alpha (8–12 Hz) and theta (4–7 Hz) and used a visual feedback paradigm. Analyses of covariance showed improvements in Avoidant Personality Accentuation within the experimental group. Our data suggest that 12 sessions of this neurofeedback intervention might be effective in reducing avoidant and stress-related personality traits in patients with alcohol use disorder.

## Introduction

Problematic personality traits including high neuroticism or impulsivity as well as co-morbid personality disorders are common in alcohol use disorder (AUD) (Ruiz et al., 2008; Lackner et al., 2013). Verheul (2001) suggested different explanations of the high comorbidity of addiction and personality disorders. These explanations are related to dysregulations in distinct neural circuitries and are defined as (1) the behavioral disinhibition pathway, (2) the stress reduction pathway, and (3) the reward sensitivity pathway. Each pathway supports the comorbidity of different DSM-IV personality disorders and substance abuse. Whereas antisocial and borderline disorders are assumed to arise via the behavioral disinhibition pathway and are associated with serotonin deficiency, the reward sensitivity pathway is most likely to account for histrionic and narcissistic disorders and is related to dopaminergic or opioidergic hyper-reactivity. By contrast, avoidant and dependent disorders are associated with stress reactivity. Stress reactivity or anxiety sensitivity might be related to increased neuronal excitability due to reduced inhibition via the GABA-glutamate receptor system (for review see Verheul, 2001). In fact, personality disorders and related traits complicate treatment and prognosis of AUD (Brorson et al., 2013).

### EEG function in AUD

Application of NF to addiction is based on findings that report altered EEG function and related brain changes in substance abusing individuals. Alterations in EEG activity were mainly observed within the alpha, theta, and beta bands (Ceballos et al., 2009). The results of most studies indicate a different synchronization and power of brain activity in alcohol dependent individuals, even in the state of abstinence. These alterations—low alpha/high beta complex—have been interpreted as the “hyperarousal” of the central nervous system which has been related to anxiety (Knyazev et al., 2004), relapse and to a worse prognosis for individuals with AUD (Saletu-Zyhlarz et al., 2004). Frontal midline theta rhythm as distinct theta activity on EEG in the frontal midline area reflects mental concentration as well as meditative state and relief from anxiety (Cavanagh and Shackman, 2015).

### Neurofeedback therapy

The impact of electroencephalography (EEG) frequency biofeedback (so-called neurofeedback) as a treatment tool in psychological conditions has been frequently investigated in recent years (e.g., Masterpasqua and Healey, 2003; Thompson and Thompson, 2007; Hammond, 2011). Neurofeedback (NF) as a modern, computer-related, operant conditioning technique involves the simultaneous measurement and retraining of brainwave patterns. It is assumed that participants learn to gain self-control over their EEG activity and that NF improves self-regulation of deficient brain patterns (Carlson-Catalano and Ferreira, 2001; Egner and Gruzelier, 2003; Hammond, 2011) which leads to a generalized enhanced self-regulation (Gevensleben et al., 2012). By utilizing signals coming directly from the central nervous system, NF has a wide range of influence on clinical conditions. Those include decrease of inattention, impulsivity, hyperactivity, stress, depressive symptoms, and anxiety (Norris et al., 2000; Vernon et al., 2004; Hammond, 2005; Thompson and Thompson, 2007; Arns et al., 2009; White and Richards, 2009; Choi et al., 2010; Sherlin et al., 2010). It is now broadly understood to practice NF in accordance to standards and guidelines (Hammond et al., 2011).

### Neurofeedback in AUD

NF as a non-pharmacological approach seems increasingly attractive in the treatment of patients with inadequate compliance and additional risk of relapse and drug abuse (Sokhadze et al., 2011). In AUD treatment, NF typically is used to change EEG frequency patterns which are related to the pathology of alcoholism. Accordingly, NF assessments to treat AUD were designed to effectively regulate deficient brain waves and pathological brain processes found in alcoholic patients. However, there exist various NF protocols for addictive disorders, some giving feedback from alpha and theta EEG frequencies, which are associated with a tranquil or calm state of mind, others focusing on sensorimotor rhythm protocol (SMR)—as a high SMR amplitude has been linked to improved attention. Likewise, combination protocols have been evaluated yet with effects on brain activity, treatment retention, mood, measures of attention and also of personality (reviewed by Sokhadze et al., 2008). Currently, the training of frontal midline theta is of key interest in NF research. Frontal midline theta is assumed to show a strong relationship with cognitive function and psychiatric symptoms (Enriquez-Geppert et al., 2014). In healthy individuals, NF effects on working memory, focused attention, creativity, and well-being have been evaluated using eyes open theta training vs. SMR training (Gruzelier et al., 2006). NF can be used either as a stand-alone intervention to modify dysfunctional brain activation patterns without explicit instruction, or as a tool to enhance cognitive and behavioral strategies.

All in all, there is a bulk of studies evaluating EEG changes and clinical outcomes in substance use disorder, however with different NF assessments and limited evidence (Sokhadze et al., 2008). Overall, the described NF protocols diverge significantly: There are protocols with eyes closed conditions which influence vigilance and EEG activity most likely in another way than eyes open approaches; Barry et al., 2007); there are studies using pre-training or combined training, making it difficult to separate NF effects from unspecific effects; moreover, EEG measurement sites differ between the studies; and limited sample sizes are often the biggest problem. Furthermore, many of the published papers lack evidence-based medical support for this proposition (case studies, conference papers, or just guides for practice based on practice).

### Changes in personality traits through alpha/theta neurofeedback

Alpha/theta training—a special variant of NF—focuses on the training of the frequency ranges alpha (8–12 Hz) and theta (4–7 Hz). In substance abuse treatment, alpha/theta training was first introduced by Peniston and Kulkosky (1989) with the aim to induce deep relaxation/stage 1 sleep when a subject has closed eyes. The original “Peniston Protocol” used an alpha/theta training intervention with auditory feedback combined with temperature training, guided imagery, and constructed visualizations. Following alpha/theta NF, alterations in alpha and theta frequency bands and reduction of beta-endorphin levels have been observed (Peniston and Kulkosky, 1989, 1990, 1991). In addition, alpha/theta training has been reported to have beneficial effects on AUD pathology including stress-related craving, fear of relapse, depressive and PTSD symptoms as well as changes in clinical personality traits (Peniston and Kulkosky, 1990, 1991).

Studies particularly focused on investigations with the Peniston Protocol on personality measures including the *Million Clinical Multiaxial Inventory Scales (MCMI)* (Peniston and Kulkosky, 1990; Saxby and Peniston, 1995), the *16-PF Personality Inventory (16-PF)* (Peniston and Kulkosky, 1990), and the *Minnesota Multiphasic Personality Inventory (MMPI)* (Peniston and Kulkosky, 1991; Scott et al., 2005). After alpha/theta training, improvements were reported on the 16-PF scales including “warm-hearted,” “intelligent,” “emotionally stable,” “socially bold,” “relaxed,” and “satisfied” (Peniston and Kulkosky, 1990). Significant improvements have also been observed after NF on the MCMI in personality patterns such as Anxiety, Avoidance, Schizoid, Passive Aggression, Borderline, Somatoform, Alcohol abuse, Psychotic thinking, and Dysthymia, with largest effects on scale 1 (DSM-III parallel: Schizoid), scale 2 (DSM-III parallel: Avoidant), scale 12 (DSM-III parallel: Anxiety), and scale 15 (DSM-III parallel: Dysthymia) (Peniston and Kulkosky, 1990). Accordingly, effects were shown in the clinical scales of the MMPI in the areas of Hypochondriasis, Depression, Hysteria, Schizophrenia, Psychasthenia, and Paranoia, with largest effects on Depression and Psychasthenia (Peniston and Kulkosky, 1991). In a replication study with the MCMI, largest effects were observed in the MCMI scale 2 (DSM-III parallel: Avoidant) and scale 12 (DSM-III parallel: Anxiety) (Saxby and Peniston, 1995). In 2005, Scott et al. demonstrated positive effects of a combined NF protocol (beta and SMR training, followed by an alpha/theta protocol) in a mixed substance abusing population on the MMPI scales Hypochondriasis, Depression, Hysteria, Schizophrenia and Social Introversion. The authors interpreted these personality changes as an indication of a lowered level of general distress or discomfort. They concluded that there may be a reduced sense of alienation and depression, as well as defensiveness through NF. In line, Raymond et al. (2005) found that healthy individuals were more composed, agreeable, and confident after alpha/theta training. Although no changes in personality were measured or observed using the *Personality Syndrome Questionnaire* in this study, the authors suggest that “normal” personality might be too robust to change within 5 weeks. Nevertheless, *t*-test showed highest pre-post changes in the composed-anxious subscale of the *Profile of Mood States* through NF in an alpha/theta group as well as in a mock feedback group. A study with the *Symptom-Checklist-Revised* could demonstrate positive effects in opioid addictive patients by the use of an combination protocol consisting of SMR training, followed by alpha-theta training. Strongest effects were evident in the Obsessive-Compulsive scale and in Psychoticism, as well as in Somatization. No effects were found in Anxiety, Phobic anxiety, Depression, and Paranoia (Arani et al., 2010). Accordingly, the authors demonstrated effects in the *General Health Questionnaire 28* on the subscales Physical Symptoms and Depression, but not on the subscales Anxiety and Social Functions (Dehghani-Arani et al., 2013). Surmeli and Ertem (2009) demonstrated the first evidence for the positive effects of quantitative EEG-guided neurofeedback training in antisocial personality disorders. Clinical improvements were shown in—amongst others—aggression, failure to sustain consistent work, insomnia, and loss of interest in life.

### Study aim

In this study, we sought to evaluate the effects of NF on personality characteristics including Clinical Personality Accentuations (PA) according to ICD-10 (Dilling et al., 1991) and DSM-IV (Saß et al., 1996) in a cohort of Austrian AUD patients treated in a therapeutic community setting. Additionally, we wanted to investigate the effects of NF with respect to the Big Five. This exploratory pilot project was designed to use a modified version of the “Peniston Protocol.” While alpha/theta training is usually done with an eyes closed auditory feedback paradigm, we aimed to introduce a new variant of alpha/theta training by using a visual feedback paradigm. We suggested that an advantage of the eyes open approach is that it may alleviate anxiety in highly anxious participants; in particular patients with anxiety disorders, who in a laboratory setting may be apprehensive about closing their eyes. Moreover, vigilance problems are common when training with eyes closed. Nevertheless, the choice for eyes open and theta at Fz has further implications than just less vigilance problems. Negative emotions such as anxiety and fear are tightly integrated with control processes implemented in the midcingulate cortex (Cavanagh and Shackman, 2015). We suggested that the neurophysiological self-regulation of the midline theta activity could be a further effect mechanism of this NF approach. In addition, we wanted to isolate the NF training from other relaxation-inducing techniques (e.g., thermal biofeedback, systematic desensitization, and autogenic instructions; Egner et al., 2002). Furthermore, we attempted to determine the reproducibility and practicality of an economical short-term NF intervention (12 sessions at most). Shortening the NF protocol and reducing the training to 6 weeks was intended to increase clinical practicability. As proposed in the literature (e.g., Peniston and Kulkosky, 1990, 1991; Saxby and Peniston, 1995; Raymond et al., 2005; Scott et al., 2005) it is very likely that alpha/theta NF acts on systems related to stress and anxiety (1) by regulating alpha and theta brain waves and (2) by activating self-management mechanisms and self-instruction processes. We therefore hypothesized that especially personality traits linked to lacking control experience and the stress reactivity pathway (avoidant and dependent traits including Neuroticism) would change through the intervention. The patients, together with the treatment procedure in this study, were the same as those contained in our previous report focussing on changes in brain activity (Lackner et al., 2015). This paper examines a different set of measures on the subjects with an elaborated focus on changes in Personality Accentuations related to the DSM-personality disorders.

## Materials and methods

### Participants

Thirty male AUD patients (15 per each group) were recruited. In the pre-test phase, five patients dropped out (*n* = 3 due to lacking motivation and intolerance of EEG conductive paste, respectively; *n* = 1 due to relapse during the therapy stay; *n* = 1 due to meeting exclusion criterion of psychosis). Therefore, a sample of twenty-five patients resulted. All study participants underwent long-term therapy (from 6 to 18 months duration; Table 1 gives means and standard deviations) at the Grüner Kreis Society, a drug rehabilitation center in Austria. The patients were treated within the setting of a therapeutic community (De Leon, 2000), which includes medical attendance, psychotherapy, and sociotherapy. The therapeutic community concept is characterized by flat hierarchical structures. Furthermore ex-users are part of the therapeutic staff. All patients were clinically detoxified in advance of therapeutic community treatment and were living drug-free within the community (excepting psychotropic medication, see Table 1) during the study. The criterion for inclusion was AUD (F10.2) diagnosed by ICD-10 (Dilling et al., 1991). Exclusion criteria were epilepsy, organic brain damage, and psychotic disorder, which were assessed from the patients' data base. Furthermore intelligence was measured by means of the Wonderlic Personnel Test (Wonderlic inc, 1999). Patients with cognitive deficits (IQ < 85) were excluded from the study. In general, patients with severe mental retardations are not accepted to enter the Grüner Kreis Society therapeutic community treatment.

**Table 1:** Means, standard deviations and percentages of demographic background, treatment history, personality disorders and medication use.

	**N**	**Age**			**Therapy duration (weeks)**			**Number of previous therapy stays**			**Personality disorders (Cluster B)**	**Antidepressant (SSRI) medication**
		***M***	***SD***	**Range**	***M***	***SD***	**Range**	***M***	***SD***	**Range**	***N***	**(Yes, %)**
EG	13	38.9	9.1	28–56	36.1	20.3	8–79	3.23	2.89	1-12	2	46.2
CG	12	40.5	8.8	27–52	55.4	40.7	2–156	2.67	2.31	1-9	2	58.3
Total	25	40.2	8.8	27–56	45.4	32.6	2–156	3.0	2.6	1-12	4	52.0

*EG, Experimental group; CG, Control group*.

The participants were allocated randomly either to the experimental group (EG, *n* = 13) or to the control group (CG, *n* = 12). The incidence of psychiatric comorbidity (affective disorders and Cluster B personality disorders) did not differ between the groups [$\chi_{\left(1\right)}^{2}$ = 0.337, *p* = 0.561]. There were also no significant differences between the groups at pre-testing for age (*t* = −0.18, ns.), therapy duration (*t* = −1.52, ns.), number of therapy stays (*t* = 0.54, ns.), medication (χ^2^ = 0.371, ns.), or any of the psychometric scales (see Table 1). All patients had pre-experience with relaxation techniques. All participants on the waiting list were asked to undergo 6-week alpha/theta training after completing test-phase. Written informed consent and approval of the ethics committee of the Medical University of Graz (EK number: 21-085 ex 09/10), in accordance with the Helsinki Declaration, were obtained. The intervention phase took place between 2010 and 2011. Sample characteristics are shown in Table 1.

### Experimental procedure

Patients were allocated randomly either to the EG or to the CG, and all participants agreed to be randomized. The randomization was done with a random number table. A computer-based psychological test battery (including personality assessments) was performed at pre- (t1) and post-test (t2) in both groups. EG participants completed 12 sessions of NF training in addition to their usual treatment program over a period of 6 weeks. Each training session typically lasted 30 min (10 min EEG montage, 20 min training phase) and the sessions were scheduled during the week at the same time each day. The control group received their usual treatment without NF intervention. All tasks took place in a soundproof and dimly lit room at the rehabilitation center. A 5-month follow-up study was performed to determine long-lasting training effects. Thirteen participants were available for follow-up testing (t3; EG: *n* = 6, CG: *n* = 7).

### Neurofeedback protocol

The group training protocol focused the augmentation of alpha and theta activity simultaneously. Traditional fixed bands were used for alpha (8–12 Hz) and theta (4–7 Hz). Feedback electrodes were placed at Fz, Cz, and Pz, the ground electrode was placed on the nasion, and a reference electrode was placed on the tip of the nose. The brain's electrical activity was displayed on a screen in the form of two bars, representing alpha and theta activity. Feedback was given for the trained frequencies, representing amplitudes greater than preset thresholds. Therefore, two bars were presented on a screen. The bar on the left side of the screen represented theta activity and the bar on the right sight represented alpha activity. The thresholds for the alpha and theta bars were adapted after each feedback run on the basis of the run immediately previous (median band power of the last 5 s of each run). Each NF session consisted of six training runs (each 180 s). Participants were instructed to enhance both bars and to keep them over a yellow marker (threshold). Successful runs (enhancement of both frequency bands) were rewarded by a smiling face; when a trial was unsuccessful an unhappy face was presented. If a participant could enhance only one frequency band (either alpha or theta), a neutral emoticon was presented. In addition to continuous feedback, reinforcement was given after the session in the form of points. The participants were instructed to find the most successful mental strategy to acquire a relaxed brain state. No other specific instructions were given. EEG data was processed using Matlab software and artifacts were removed manually. The details of the EEG acquisition and the precise technical background of NF application are described in our previous paper (Lackner et al., 2015).

### Personality inventories

The Inventory of Clinical Personality Accentuations (ICP) by Andresen (2006) is a 132-items questionnaire and comprises 11 scales measuring the criterion-based contents of DSM-IV axis II disorders (Paranoid, Schizoid, Schizotypal, Antisocial, Borderline, Impulsive-explosive, Histrionic, Narcissistic, Avoidant, Dependent, Obsessive). The 4-point Likert scales range from “completely wrong” to “completely right.” The ICP was developed based on the dimensional view of classification (instead of the categorical view) in accordance to an 8-Factor-Model (Basic-Eight-Questionnaire). Thus, the word “personality accentuation” (PA) is used instead of “personality disorder.” There is a high internal comorbidity, as well as with personality disorders or other axis-I-disorders (e.g., substance use disorder or obsessive-compulsive disorder). Comparison studies exist, suggesting good external and discriminant validity and the internal consistency of the ICP scales ranges from 0.76 to 0.92. Correlations have been found with other dimensional clinical personality inventories e.g., the PSSI by Kuhl and Kazén (1997) or the CATI by Coolidge (1993) and Andresen (2006).

The German version of the *NEO-Five-Factor Inventory (NEO-FFI)* by Borkenau and Ostendorf (2008) was used to assess the Big Five personality traits Neuroticism, Extraversion, Openness to Experience, Agreeableness, and Consciousness. Satisfactory test-retest reliabilities (*r* = 0.71 and *r* = 0.82) and internal consistency reliabilities (α = 0.72 and α = 0.87) are available. Accordingly, the NEO-FFI presents good factorial and discriminant validity (McCrae and Costa, 2004).

After 6 weeks of training, an *evaluation protocol* was administered including a subjective rating of training success (“*How do you rate your training success across the last 6 weeks?*”) and rating of mood improvement (“*How did your mood improve through neurofeedback training?*”). The responses were evaluated by a six-point rating scale ranging from one (*absolutely no improvement)* to six (*very much improved*).

### Statistical analyses

Analyses of co-variance (ANCOVAs) with Group as a between-subject factor, Test-score at post-test as a within-subject factor, and Test-score (rank) at pre-test as covariate were performed. Paired sample *t*-tests were performed for *post hoc* evaluations within the group. Additionally, the false discovery rate (FDR) was used as correction for multiple comparisons (Benjamini and Hochberg, 1995). A Wilcoxon-Mann-Whitney test was performed to compare differences in decreases and increases in Avoidant PA scores of the participants. The follow-up data were analyzed using repeated-measures analyses of variance (ANOVAs).

## Results

For NEO-FFI, raw scores were transformed to T-scores (*M* = 50, *SD* = 10) based on normative data for males between 30 and 49 years, as presented in the manual (Borkenau and Ostendorf, 2008). For ICP, no appropriate normed scores were available and raw scores were analyzed. However, in order to facilitate interpretation of the results, we calculated *post hoc t*-tests to compare the obtained ICP-scores with scores of the general population.

### Changes in clinical personality accentuations

**Figure 1:**
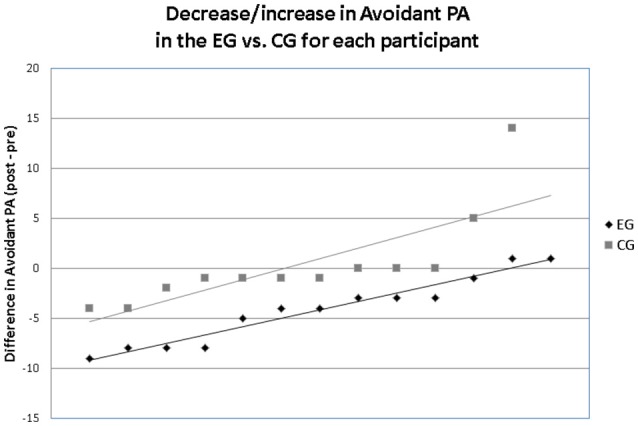
Changes from pre- to post-test of each patient.

ANCOVAs indicated a significant effect of NF on the ICP scale Avoidant PA. Trending statistical effects were found in the Schizoid PA, Schizotypal PA, and Narcissistic PA (*p* < 0.10). Paired sample *t*-tests indicated a significant decrease in Avoidant PA and Schizotypal PA in the EG, while no changes in these scales were observed in the CG. Table 2 gives the statistics, means, and standard deviations for the EG and the CG at pre- and post-test for all ICP scales. After correction for multiple comparisons using the FDR (Benjamini and Hochberg, 1995), no *F*-test achieved statistical significance with the exception of the Avoidant PA. A descriptive evaluation of each subject is displayed in Figure 1, demonstrating the individual changes (pre-test minus post-test) in Avoidant PA. It has been shown that in the EG 85% of participants decreased in Avoidant PA, whereas in the CG only 58% decreased. In the EG the difference between post- and pre-test was higher (the majority decreased >4 raw score points) than in the CG (according to Wilcoxon-Mann-Whitney, *U* = 31.00, *p* = 0.018; see Figure 1).

**Table 2:** Differences in ICP and NEO-FFI scores between the groups (Means, standard deviations, *F*-statistics and effect sizes for ANCOVAs, *p*-values for paired sample *t*-tests).

	**EG** ***Means (**±**SD)***		**CG** ***Means (**±**SD)***					
	**Pre**	**Post**	**Pre**	**Post**	***F-value***	**Eta squared**	**EG *t* score**	**CG *t* score**
**ICP**								
Paranoid	26.15 (3.98)	24.92 (6.96)	26.45 (5.68)	25.27 (6.39)	0.047	0.003	0.70	0.89
Schizoid	24.92 (5.12)	23.31 (4.29)	23.18 (5.56)	24.27 (5.31)	2.98	0.142	1.48	−1.20
Schizotypal	22.15 (6.59)	19.54 (6.84)	18.55 (4.89)	19.36 (4.57)	3.31	0.150	2.64^*^	−1.53
Antisocial	19.92 (6.13)	19.38 (5.33)	19.73 (5.39)	20.91 (6.47)	0.596	0.030	0.43	−0.844
Borderline	21.46 (6.58)	20.23 (5.00)	19.55 (4.97)	19.64 (4.80)	0.024	0.002	1.43	−0.08
Impulsive-explosive	24.08 (7.43)	22.31 (7.57)	25.0 (5.92)	24.45 (5.54)	0.176	0.011	1.09	0.53
Histrionic	22.62 (6.32)	21.23 (5.69)	24.2 (5.16)	22.8 (3.77)	0.086	0.014	1.12	1.80
Narcissistic	21.92 (7.32)	19.54 (4.39)	22.91 (7.05)	22.0 (5.44)	2.20	0.110	2.13	0.70
Avoidant	25.54 (6.25)	21.38 (5.81)	22.09 (5.82)	22.55 (5.41)	4.46^*^	0.187	4.45^**^	−0.30
Dependent	25.46 (5.70)	22.92 (7.15)	21.09 (4.97)	22.55 (4.12)	1.45	0.065	1.80	−1.06
Compulsive	26.54 (4.86)	23.46 (4.47)	28.18 (3.89)	25.55 (4.25)	0.213	0.019	2.78^*^	2.63^*^
**NEO-FFI**								
Neuroticism	59.77 (9.82)	53.54 (12.42)	54.58 (4.96)	59.92 (4.34)	1.52	0.065	2.68^*^	1.27
Extraversion	47.33 (9.75)	50.62 (11.17)	50.67 (7.10)	52.08 (5.43)	0.089	0.004	−1.72	−0.929
Openness	41.77 (11.61)	41.54 (7.48)	47.50 (7.25)	47.58 (9.28)	0.899	0.039	0.113	−0.052
Agreeableness	49.00 (6.28)	48.62 (12.24)	47.73 (6.07)	49.27 (7.31)	0.380	0.018	0.144	−1.05
Conscientiousness	51.00 (13.17)	52.54 (12.38)	53.45 (11.16)	56.27 (9.90)	0.199	0.009	−0.730	−1.48

Significant effects

* *p < 0.05*,

** *p < 0.01 in bold letters; EG, Experimental group, CG, Control group; ICP, Inventory of Clinical Personality Accentuations; NEO-FFI, NEO-Five-Factor Inventory*.

Follow-up data revealed that after 5 months, the effects of NF on Avoidant PA remained almost stable. The repeated measures ANOVA indicated a time × group effect by trend [*F*_(10, 2)_ = 3.11, *p* = 0.065; EG: *M*_*t*1_ = 25.54, *SD*_*t*1_ = 6.25; *M*_*t*2_ = 22.0, *SD*_*t*2_ = 3.51; *M*_*t*3_ = 23.14, *SD*_*t*1_ = 4.45; CG: *M*_*t*1_ = 23.0, *SD*_*t*1_ = 6.90; *M*_*t*2_ = 25.33, *SD*_*t*2_ = 4.13; *M*_*t*3_ = 22.67, *SD*_*t*1_ = 5.68]. *Post hoc t-*tests in the EG showed higher Avoidant PA in the pre-test compared to follow-up [*T*_(6)_ = 4.35, *p* = 0.005]. No significant difference was observed between post-test and follow-up [*t*_(6)_ = −1.33, *p* = 0.231].

### Changes in big five personality traits

There were no significant ANCOVA differences for the Big Five personality traits. Paired sample *t*-tests revealed significant improvements in Neuroticism in the EG. However, the FDR showed no statistical significance (see Table 2).

### Subjective rating of training success

Subjective rating of training success increased over the sessions (*t* = −2.28, *p* < 0.05; *M*_*SS*1_ = 4.42, *SD*_*SS*1_ = 1.44; *M*_*SS*12_ = 5.17, *SD*_*SS*12_ = 1.03). At the end of training, 31% of the patients rated their overall training success with six points (“very well”), 15.4% with five, and 46.2% with four points. All EG patients reported improvements in mood over the 6 training weeks, with 15.4% reporting strong improvements. The participants were afterwards asked about the applied mental strategy. Almost all participants reported using cognitive strategies to achieve a relaxed state.

## Discussion

This study was performed to determine if subjects with AUD would benefit from NF treatment using an eyes-open feedback paradigm. The focus of this research was based on the potential change in Clinical PA according to DSM-IV and ICD-10. Our data showed that a 6 week NF intervention had a positive effect on Avoidant PA. However, there were no changes in other PAs or in global Big Five personality dimensions after NF training. These results will be discussed further in detail.

As shown in fMRI studies, NF has positive effects on the self-control functions of the brain (Johnston et al., 2010). It is estimated that NF enhances self-efficacy (e.g., Carlson-Catalano and Ferreira, 2001; Thompson and Thompson, 2007; Linden et al., 2012) due to the experience of success on regulating one's own brain activity. Additionally, the increased alpha activity is assumed to support the patient to be calm and to better tolerate stress (Knyazev et al., 2004; Thompson and Thompson, 2007). The modulation of frontal midline theta (as one effect of the present study; see Lackner et al., 2015) has been associated with cognitive processes, meditative states and reduction of anxiety before (Enriquez-Geppert et al., 2014; Cavanagh and Shackman, 2015).

By gaining control over physiological processes, it is likely that participants additionally gain more self-confidence and reduce emotional stress, feelings of inadequacy, insecurity, and fear. These traits are characteristic DSM-criteria for avoidant personality disorder which is also known as anxious personality disorder (Saß et al., 1996). Persons scoring high on avoidant personality traits are affected by social stress and often do not feel able to cope with social demands (Andresen, 2006). According to Verheul (2001), avoidant personality disorders in AUD patients may arise via the stress reactivity pathway. This pathway predicts that individuals scoring high on traits related to anxiety and mood instability seem to be more vulnerable to stressful life-events. It is well established that besides genetically-predisposed neuro-physiological and neurochemical patterns, the most frequently reason to use alcohol is to reduce tension and stress (Khantzian, 1997). Therefore, self-medication can become a strong motive for substance use in highly anxious individuals. In this context, NF as a procedure to reduce stress and anxiety (Thompson and Thompson, 2007) could interfere with the stress reactivity pathway by impacting related brain systems directly. Initial findings with the Peniston Protocol showed that NF significantly lowered clinical scales, with highest effects on scales related to anxiety, abnormal fears, tension, social avoidance, obsessive-compulsive symptoms, depression, and self-criticisms (MMPI: Psychasthenia, Depression; Peniston and Kulkosky, 1991; MCMI: Schizoid, Avoidant, Anxiety, Dysthymia; Peniston and Kulkosky, 1990). In agreement with our findings, Saxby and Peniston (1995) reported the biggest effects of alpha/theta training in a DSM-III equivalent of Avoidant Personality, scale 2 of the MCMI. Accordingly, Scott et al. (2005) reported decreases in MMPI scales known as neurotic triad (Hypochondria - Hs, Depression - D, Hysteria - Hy), which was interpreted as lowered level of general distress. Consequently, the current study confirms previous results of traditional alpha/theta eyes-closed training albeit with another methodology. As by Raymond et al. (2005), who observed more confidence and a better mood in experimental participants than in controls, less shyness and more self-esteem (denoted amongst others by Avoidant PA) was demonstrated in this experiment too. However, Raymond et al. showed anxiety improvements through NF, independently whether real feedback or placebo feedback was given. Hence, it could be possible that the improved self-efficacy expectation could be the key factor of efficacy of NF therapy as proposed by Carlson-Catalano and Ferreira (2001). It is well known that NF enables the patient to gain more control over physiological processes which could increase self-efficacy.

Similar to Avoidant PA, Neuroticism involves negative emotionality and physiological reactivity to stress (McCrae and John, 1992). Although there were changes in the EG from pre- to post-test, the ANCOVA did not reached statistical significance. We assume that NF affects personality at a highly specific level, but it is not that easy to change global personality structure with NF. Nevertheless, most previous studies used a repeated-measures design, whereas the present study applied a more stringent ANCOVA model. This could be another reason why traits as Neuroticism or others which have been found relevant before—like schizoid or depressive traits (Peniston and Kulkosky, 1990)—were not statistically significant in this study.

In contrast to Arani et al. (2010) and Dehghani-Arani et al. (2013), respectively, who failed to show NF effects on anxiety, and who concluded that there is a need of 40–50 sessions of alpha/theta training to improve anxiety, we found that 12 sessions of our new NF method could be enough to decrease anxiety-related traits. However, experts argue about the difference between alpha/theta training and conventional relaxation techniques, hypnosis, and meditation procedures (Egner et al., 2002). Probably, alpha/theta NF with closed-eyes can be understood as a highly sophisticated relaxation or trance technique. By using an eyes-open approach, we wanted to avoid on the one hand the problem of vigilance. On the other hand, other brain processes (e.g., midline theta) can be trained with opened eyes. We believe that NF with eyes-open can be much more specific than other conventional treatment techniques; EEG curves are easy to monitor for the NF trainer and the changes in brain activity can be displayed objectively on the screen for the patient. This might be an improvement over other therapeutic or stress reduction techniques. In general, NF effects are less dependent on therapist-client-interaction, which may result in NF to be a preferred treatment in substance use disorders. However, NF application requires time to learn and varies depending on the initial condition that the patient starts with (Gunkelman and Johnstone, 2005).

In this paper, no completely new experiment was conducted; nevertheless, the NF effects on different variables of the main study were investigated. In our recent paper which focused on the effects on brain activity and symptoms related to AUD, EEG effects from pre- to post-test through the NF training were observed. Participants reported increased control of the EEG and trending changes in the midline alpha and theta band were found (Lackner et al., 2015). However, personality variables investigated here were not related to changes in brain activity.

### Limitations

Even though the participants were asked about the applied mental strategy afterwards, we cannot distinguish between effects of self-regulation of the brain and the strategies participants used to achieve these changes. Another problem inherent in a study of this kind is that the experimental participants received extra contact and attention as part of their treatment. Therefore, non-specific effects of augmented attention, therapist interaction, or expectancies cannot be excluded. These effects might be disentangled by protocols utilizing placebo treatment, or even better, an active control group receiving another training protocol. Considering the fact that we conducted a proof-of-principle study using such a feedback paradigm for the first time, the ethical justification for the use of placebo feedback was not lacking. Hence, we considered a waiting control group as appropriate. In future studies, the best option would be the comparison with an active control group as Monastra et al. (2002) could show. As half of the participants were taking SSRIs, medication effects could have influenced the results. However, we conducted *post hoc* ANCOVAs with medication as covariate and did not find this to be a significant factor. Although all patients were in the recovery stage, we had no data about time elapsed since detoxification. The study was further limited due to the fact that neurological exclusion criteria (e.g., epilepsy, organic brain damage) were evaluated simply through anamnesis. Moreover, there was a high range of median therapy durations. Future studies should include medication-naïve AUD patients or at least individuals at a similar stage of treatment. In general, larger study samples are urgently needed in NF research.

### Clinical implications

In contrast to prior research with alpha/theta feedback in addiction treatment (Saxby and Peniston, 1995; Scott et al., 2005), this study used a visual alpha/theta training paradigm. This was done because in previous clinical experience, we observed that most patients were reluctant to close their eyes. The NF intervention under investigation was especially developed to treat highly anxious patients, who in a laboratory setting may be apprehensive about closing their eyes. Furthermore, the training was intended to be rather short (12 sessions at most), which should increase clinical practicability. Additional training tools as originally applied by Peniston and Kulkosky (1989, 1990, 1991) were also omitted in the present study.

In summary, the changes on Avoidant PA give an indication that the mechanism of visual alpha/theta NF may allow participants to better tolerate anxiety eliciting situations. In clinical practice, NF application can be seen as a useful additional therapy tool, which can improve the patient's self-efficacy and reduce feelings of insecurity. As a result, the management of patients could be facilitated in the therapeutic community. Especially Avoidant PA appears to be more sensitive to NF than other PA's in all patients. In order to show how clinically meaningful a reduction of 4.16 pt. in Avoidant PA is, we performed *post hoc* one-sample *t*-tests. The findings indicated that the significant difference between the investigated patients from the EG and healthy control persons (*M* = 19.47) at pre-test [*t*_(13)_ = 3.50, *p* < 0.01] disappeared at post-test [*t*_(13)_ = 1.19*, p* = 0.26]. Follow-up data refer to a longer-lasting effect of personality change; however these data are rather limited.

From a clinical perspective, we observed that the intervention was accepted well and that the patients appreciated the opportunity to be treated with NF. This was supported by the self-ratings. However, as in most cases of therapeutic intervention, NF treatment alone might not be sufficient to achieve optimal clinical outcome. On basis of the existing literature and our own observations, we recommend the implementation of NF as a common treatment tool in a multimodal addiction treatment programme.

## Conclusions

The combination of standard treatment with 6 weeks of NF training was associated with a significant decrease in Avoidant PA in male patients with AUD. We conclude that such a NF training, probably acting via neuroregulation of the brain (alpha, theta) and via improved self-management strategies, has an effect on a specific level of personality related to anxiety, insecurity, and self-consciousness. Thus, the use of this NF training could potentially impact the stress reactivity pathway and reinforce personality traits related to stress exposure. We believe that NF has the potential to enable the patient to be more self-determining and to decrease feelings of insecurity and social stress. However, research with bigger samples is needed for further evidence. Overall, NF, as a non-pharmacological treatment, could be a promising supplementary tool for addiction therapy and is practicable in therapeutic community settings. The findings are promising and may stimulate further research into the efficacy of neuro-therapeutic approaches in AUD.

## Author contributions

All authors listed have made a substantial, direct and intellectual contribution to the work, and approved it for publication.

### Conflict of interest statement

The authors declare that the research was conducted in the absence of any commercial or financial relationships that could be construed as a potential conflict of interest.
